# Case Report: Ovarian fibroma: typical presentation with Meigs’s Syndrome

**DOI:** 10.12688/f1000research.122368.1

**Published:** 2022-07-22

**Authors:** Dipesh Upreti, Dipesh Kumar Rohita, Shailendra Kumar Yadav, Niku Thapa

**Affiliations:** 1 Manipal College of Medical Sciences, Pokhara, 33700, Nepal; 2 BP Koirala Institute of Health Sciences, Dharan, 56700, Nepal

**Keywords:** Ascites, fibroma, oophorectomy, pleural effusion

## Abstract

Meigs’s syndrome is characterized by a triad of ovarian fibroma, ascites, and pleural effusion which can be managed surgically. Pleural effusion and ascites are usually transudative. Ovarian fibroma is an uncommon tumor. We herein report a case of Meigs’s syndrome in a 61-year-old woman who presented with complaints of abdominal pain for two-three months along with decreased appetite and constipation. On examination, there was decreased air entry in the right side of the chest, generalized abdominal distention, and a firm irregular mass was felt which was mobile and extending from upper border of symphysis pubis to just above the umbilicus on abdominal palpation. Chest X ray showed right sided pleural effusion, ultrasonogram (USG) abdominal and pelvis showed gross ascites, and a very large complex right ovarian cyst was confirmed by computed tomography (CT) scan. She underwent staging laparotomy with total abdominal hysterectomy, bilateral salpingo-oophorectomy and omental resection for biopsy. Biopsy showed right ovarian fibroma.

## Introduction

Meigs syndrome is defined as the triad of benign ovarian tumor, especially ovarian fibroma with ascites and pleural effusion that resolves after tumor resection. ^1^ It occurs as a result of increased capillary permeability thought to be a result of vascular endothelial growth factor (VEGF) production. Pleural effusions are usually right-sided because the transdiaphragmatic lymphatic channels are larger in diameter on the right. ^2^ The pleural effusion as well as accompanying ascites are typically transudative. Meigs syndrome is also seen in cases like large, cystic leiomyomas or other benign ovarian tumors, thecoma, cystadenoma or granulosa cell tumor. ^3^ Ovarian fibromas constitute 2 to 5% of all ovarian tumors and Meigs syndrome occurs in just 1% of these tumors indicating rarity of this clinical condition. ^4^ Though diagnosis is possible preoperatively with ultrasound and magnetic resonance imaging (MRI), a high index of suspicion may be important as it radiologically and clinically mimics ovarian malignancy. ^5^ This case is described for its rarity in presentation and clinical confusion in diagnosis.

## Case report

This was a case of 61-year-old, post-menopausal, Asian housewife, para four living three woman, presenting with abdominal distension for two to three months along with decreased appetite and constipation. She had a history of abdominal mass suspected of being ovarian malignancy for two years prior, for which she had not undergone any treatment due to personal difficulties. She experienced no vomiting and abdominal pain. She was former smoker and a known case of diabetes mellitus.

On examination, general condition was fair with no pallor, edema, lymphadenopathy or any signs of dehydration. Vitals were stable. On respiratory examination, decreased air entry was observed on the right side. Cardiovascular examination was normal. On abdomen examination, an irregular firm mass could be felt which was mobile and extending from the upper border of symphysis pubis to just above the umbilicus. The mass was mobile and non tender on palpation. Speculum examination showed normal cervix with rectocele. Vaginal examination showed an anteverted uterus with fullness felt in all the fornices separate from uterus.

**Figure 1.**
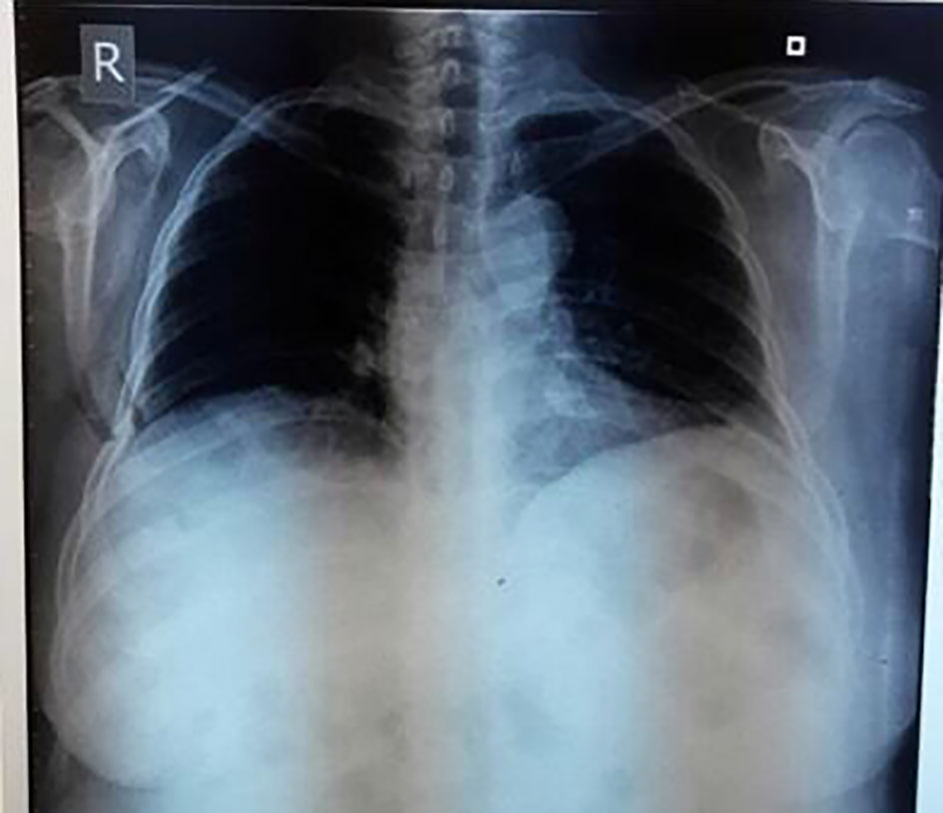
Chest X-ray showing right sided pleural effusion.

**Figure 2.**
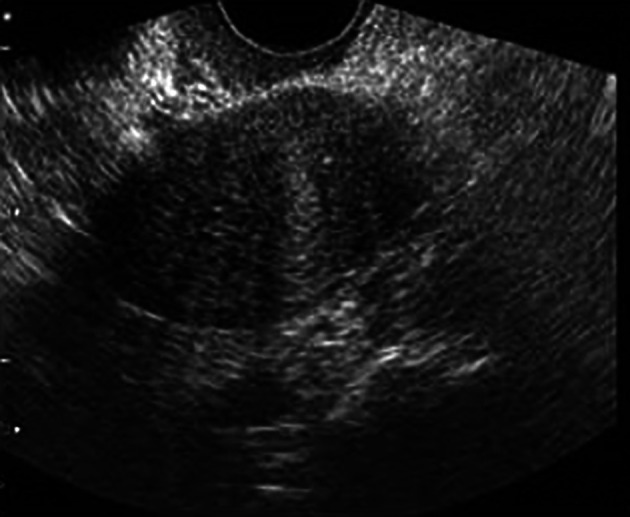
Ultrasound of the abdomen and pelvis showing ovarian cyst and ascites.

**Figure 3.**
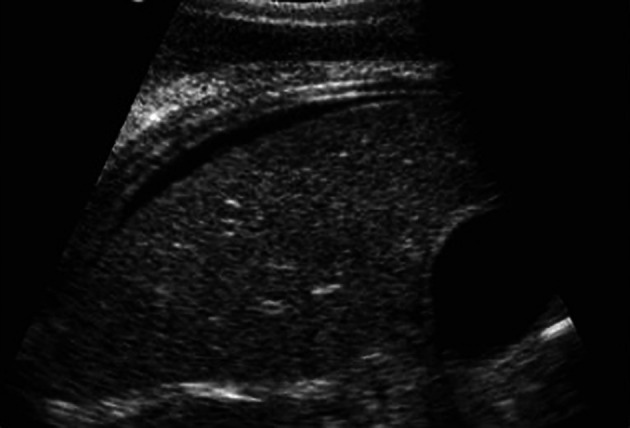
Ultrasound of the abdomen and pelvis showing ovarian cyst and ascites.

**Figure 4.**
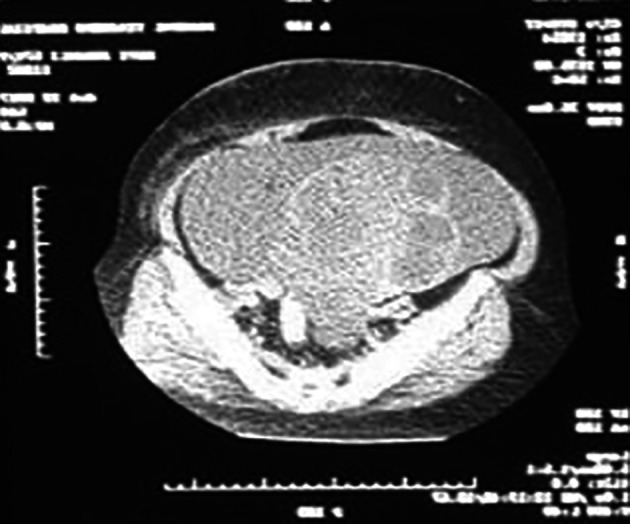
CT scan of the abdomen and pelvis showing the complex ovarian cysts.

**Figure 5.**
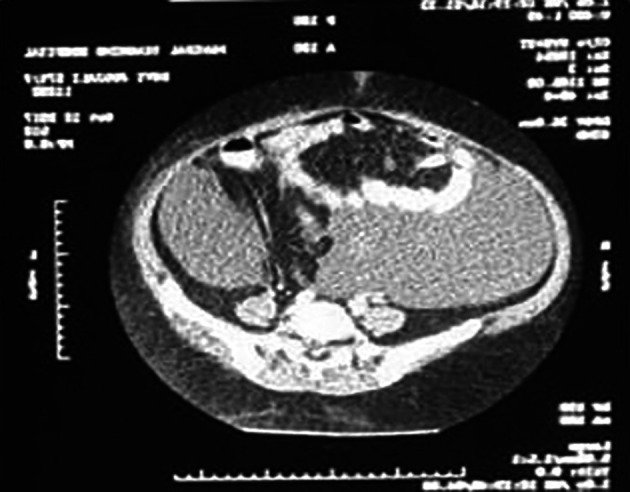
CT scan of the abdomen and pelvis showing the complex ovarian cyst.

Chest X-ray showed blunting of right costophrenic angle suggesting pleural effusion ( Figure 1). Ultrasound of abdomen and pelvis revealed gross ascitis, huge complex right ovarian cyst ( Figures 2, 3), confirmed by CT abdomen and pelvis as malignant ovarian tumor ( Figures 4, 5). Ascitic tapping showed transudative nature of fluid, cancer antigen 125 (CA-125) was 84.6 U/ml and carcinoembryonic antigen (CEA) and lactate dehydrogenase (LDH) were normal.

**Figure 6.**
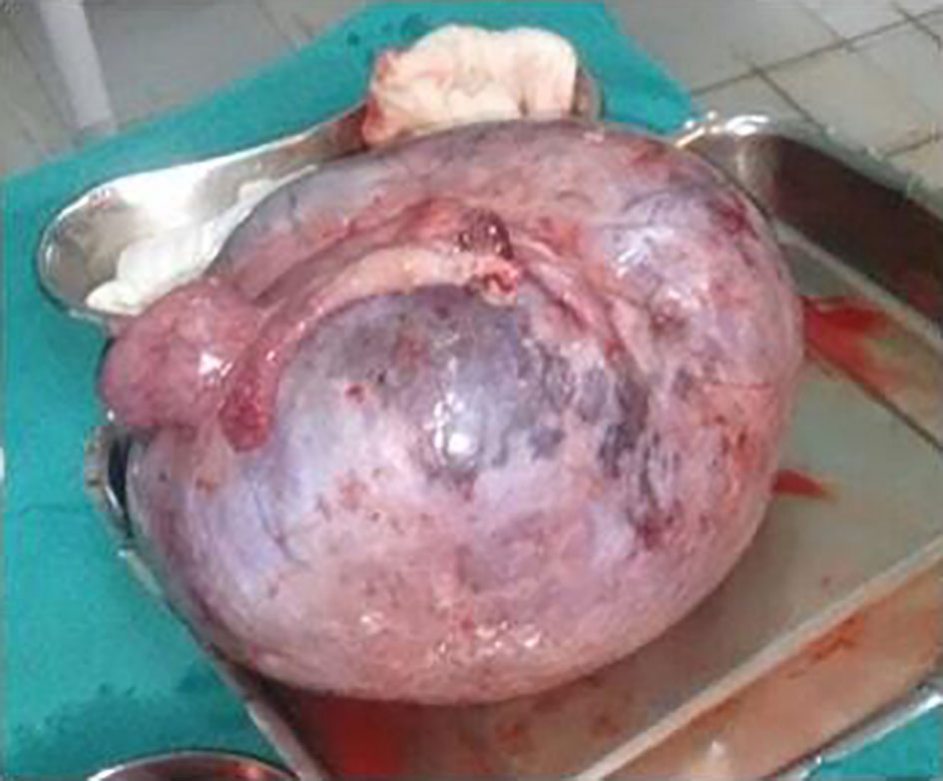
Gross surgical specimen showing right ovarian hard mass with irregular surface of 15 × 10 cm ^2^.

**Figure 7.**
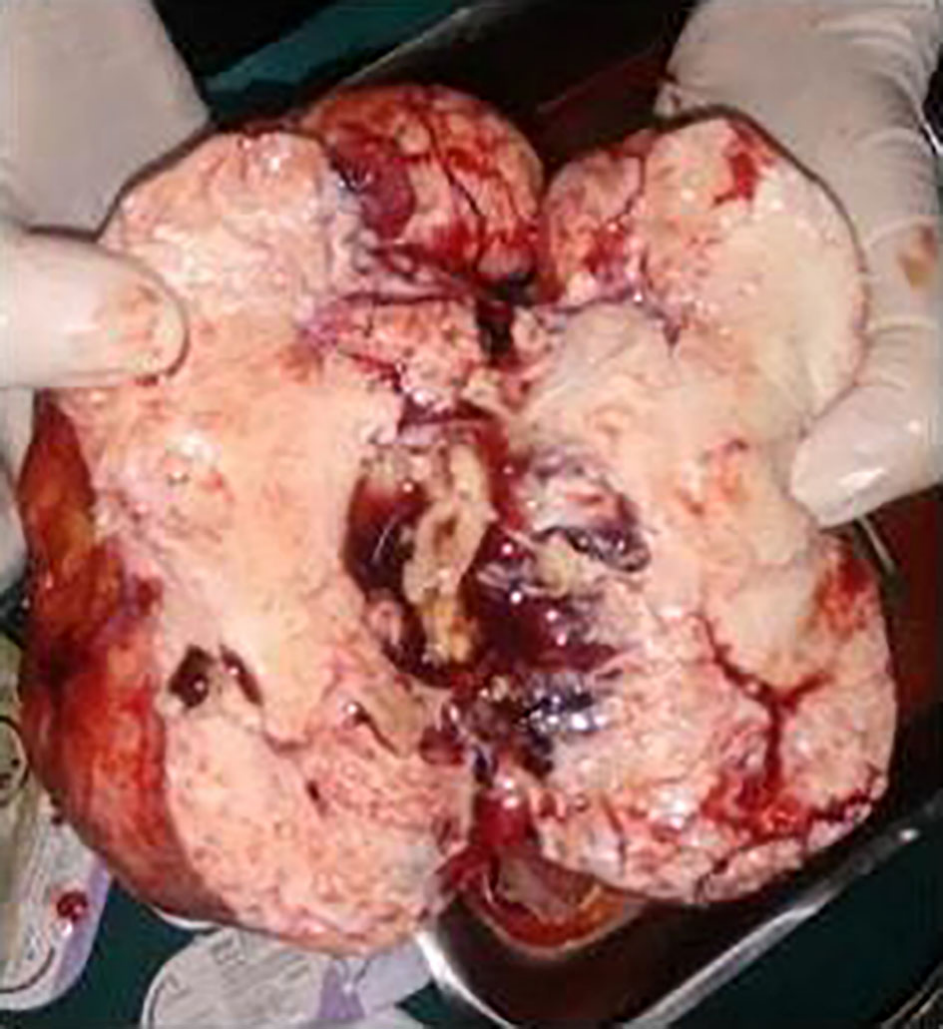
Gross surgical specimen showing right ovarian hard mass with irregular surface of 15 × 10 cm ^2^.

She underwent staging laparotomy with total abdominal hysterectomy, bilateral salphingo-oophorectomy and omental resection for biopsy. There were six litres of straw-colored ascitic fluid with right ovarian hard mass with irregular surface of 15 × 10 cm on laparotomy ( Figures 6, 7). Uterus, left ovary and cervix were normal. Omentum, bowel, liver, and peritoneal surface were normal. She was diagnosed with stage I C ovarian tumor. Her post-operative period after laparotomy with total abdominal hysterectomy and bilateral salphingo-oopherectomy was uneventful Biopsy report showed right ovarian fibroma. She recovered well and was living a comfortable life on follow up.

## Discussion

Ovarian fibromas are the most common hormonally inactive sex cord stromal tumor variants that usually occur in perimenopausal and menopausal women. ^6^ They represent only 4% of all ovarian neoplasms and are the least common major subtype of ovarian cancer. Meigs’s Syndrome occurs in just 1% of these cases. Although Meigs’s Syndrome is extremely rare, it is known to produce pleural effusion and ascites. Because several conditions are linked to the development of these common indications, the correct diagnosis and treatment are frequently missed. ^7^ The cause of Meigs’s condition is still unknown. Ascites are a common symptom of ovarian tumors, and numerous causes have been proposed, including tumor torsion and restriction of venous drainage. According to laboratory investigations, the fluid collected in most but not all cases is transudate. The chest and abdomen fluids are identical in all patients. ^8^ Peritoneal cytology, tumour markers, and other signs of malignant pathology may be confusing. Hence, laparotomy is essential for the correct identification of ovarian tumours. ^9^ Due to the rarity of this condition, this is a diagnosis of exclusion but should be considered as soon as ovarian malignancy is excluded. The presentation in our case was similar where a long-standing ovarian mass presented with ascitis and pleural effusion.

## Conclusions

Ovarian fibromas are uncommon sex cord stromal tumor commonly seen in post menopausal women. Ovarian fibroma could be a possibility in cases of ovarian tumors with ascitis and pleural effusion, especially when longstanding.

## Patient consent

Written and informed consent was obtained from the patient for the purpose of publication. The patient consented to their data being published.

## Data availability

All data underlying the results are available as part of the article and no additional source data are required.

## References

[1] Saha S Robertson M : Meigs’ and Pseudo-Meigs’ syndrome. *Australas J Ultrasound Med.* 2012 Feb;15(1):29–31. 10.1002/j.2205-0140.2012.tb00140.x 28191137 PMC5025132

[2] Krenke R Maskey-Warzechowska M Korczynski P : Pleural Effusion in Meigs’ Syndrome—Transudate or Exudate? *Medicine (Baltimore).* 2015 Dec 1;94:e2114. 10.1097/MD.0000000000002114 26656338 PMC5008483

[3] Liao Q Hu S : Meigs’ Syndrome and Pseudo-Meigs’ Syndrome: Report of Four Cases and Literature Reviews. *J Cancer Ther.* 2015;06(04):293–298. 10.4236/jct.2015.64032

[4] Mohammed SA Kumar A : Meigs Syndrome. *StatPearls.* Treasure Island (FL): StatPearls Publishing;2022 [cited 2022 May 21]. Reference Source

[5] Rouzier R Berger A Cugnenc PH : Meigs’ syndrome: is it possible to make a preoperative diagnosis? *J Gynecol Obstet Biol Reprod (Paris).* 1998 Sep;27(5):517–522. 9791579

[6] Horta M Cunha TM : Sex cord-stromal tumors of the ovary: a comprehensive review and update for radiologists. *Diagn Interv Radiol.* 2015;21(4):277–286. 10.5152/dir.2015.34414 26054417 PMC4498422

[7] Murayama Y Kamoi Y Yamamoto H : Meigs’ syndrome mimicking heart failure with preserved ejection fraction: a case report. *BMC Cardiovasc Disord.* 2020 Oct 7;20(1):436. 10.1186/s12872-020-01718-4 33028203 PMC7542734

[8] Latta RJ Lee PDK : Meigs’ syndrome in a young woman. *J Adolesc Health Care.* 1981 Jun 1;1(4):313–315. 10.1016/S0197-0070(81)80012-5 7333935

[9] Abad A Cazorla E Ruiz F : Meigs’ syndrome with elevated CA125: case report and review of the literature. *Eur J Obstet Gynecol Reprod Biol.* 1999 Jan 1;82(1):97–99. 10.1016/S0301-2115(98)00174-2 10192495

