# Supplementary material for: Case Report: Ovarian fibroma: typical presentation with Meigs’s Syndrome
Source: F1000Res. 2022 Jul 22;11:815. [Version 1] doi: 10.12688/f1000research.122368.1 (PMC11137481; doi:10.12688/f1000research.122368.1)
Supplement: Peer review history

# Reviewer report: Reviewer response for version 1

**DOI:** 10.5256/f1000research.134349.r233449

**Published:** 2024-05-29

**Authors:** Heera Shenoy

**Affiliations:** 1 OBG, Malabar Medical College Hospital and Research centre, Kozikhode, Kerala, India; 2 Malabar Medical College Hospital and Research Centre, Kozhikode, India

The abstract has been written well. The case presentation is in detail and well described. Images are apt and adequate.Discussion is short and could have been elaborated including pathophysiology, differential diagnosis and the options with counselling part. Pseudo meigs and the investigative approach could have been included and the clinical orientation need be established.

Are enough details provided of any physical examination and diagnostic tests, treatment given and outcomes?

Yes

Is the case presented with sufficient detail to be useful for other practitioners?

Yes

Is sufficient discussion included of the importance of the findings and their relevance to future understanding of disease processes, diagnosis or treatment?

Yes

Is the background of the case’s history and progression described in sufficient detail?

Yes

Reviewer Expertise:

endocrine

I confirm that I have read this submission and believe that I have an appropriate level of expertise to confirm that it is of an acceptable scientific standard.

# Reviewer report: Reviewer response for version 1

**DOI:** 10.5256/f1000research.134349.r262936

**Published:** 2024-05-07

**Authors:** Bijal Patel

**Affiliations:** 1 The Gujarat Cancer and Research Institute, Ahmedabad, Gujarat, India

**Case report Review**

****

**Abstract**

 1. Biopsy showed right…..

 Query - Do you mean the final histopathology ?

**Case report**

 2. Examination Ascitic fluid showed transudative….

 Query - Kindly mention:

 - Was ascitic fluid sent for cytology?

 - Was a pleural fluid tapping done and sent for cytology?

 - Why was LDH done among the tumor markers?

 3. She was diagnosed with stage IC ovarian tumor…..

 Query - How has this stage been assigned?

 4. Biopsy report showed right ovarian fibroma….

 Query

 - Was it the final Histopathology report?

 - Kindly elaborate whether this diagnosis was made on the gross and microscopic features … if so please include the microscopic pictures also and also mention description of microscopy including the atypical cells and mitotic rate.

 - Was any immunohistochemistry done… if so please mention.

**Discussion**

 5. Laparotomy is essential for correct…..

 Query - Pathological diagnosis also plays a role… kindly discuss on those features and how it can be differentiated from fibrosarcoma and mitotically active ovarian fibroma

Are enough details provided of any physical examination and diagnostic tests, treatment given and outcomes?

Yes

Is the case presented with sufficient detail to be useful for other practitioners?

Yes

Is sufficient discussion included of the importance of the findings and their relevance to future understanding of disease processes, diagnosis or treatment?

Yes

Is the background of the case’s history and progression described in sufficient detail?

Yes

Reviewer Expertise:

Gynecologic Oncology

I confirm that I have read this submission and believe that I have an appropriate level of expertise to confirm that it is of an acceptable scientific standard.

# Reviewer report: Reviewer response for version 1

**DOI:** 10.5256/f1000research.134349.r233450

**Published:** 2024-03-04

**Authors:** Irene Iavarone

**Affiliations:** 1 Department of Woman, Child and General and Specialized Surgery, University of Campania “Luigi Vanvitelli”, Naples, Italy

I read with great interest the present Manuscript which falls within the aim of the Journal. In my honest opinion, the topic is interesting enough to attract the readers’ attention. Methodology is accurate and conclusions are supported by the data analysis. Nevertheless, authors should clarify some points and improve the discussion citing relevant and novel key articles about the topic. For all those reasons, I suggested performing the minor revisions.

 - Authors may specify whether they performed either peritoneal or pleural cytology;

 - Please clarify the Follow-Up time in the Case Report section;

 - In Discussion, Authors should parallel their findings with novel evidence about the topic. Please consider: (ref-1)

 - The Authors have not adequately highlighted the strength and limitations of the study. I suggest better specifying those points.

Are enough details provided of any physical examination and diagnostic tests, treatment given and outcomes?

Partly

Is the case presented with sufficient detail to be useful for other practitioners?

Yes

Is sufficient discussion included of the importance of the findings and their relevance to future understanding of disease processes, diagnosis or treatment?

Partly

Is the background of the case’s history and progression described in sufficient detail?

Partly

Reviewer Expertise:

Gynecologic oncology, gynecology, endometriosis, Reproductive Medicine

I confirm that I have read this submission and believe that I have an appropriate level of expertise to confirm that it is of an acceptable scientific standard, however I have significant reservations, as outlined above.

## References

[1] : Meigs Syndrome and Elevated CA-125: Case Report and Literature Review of an Unusual Presentation Mimicking Ovarian Cancer. *Medicina (Kaunas)* .2023;59(9) : 10.3390/medicina59091684 10.3390/medicina59091684 37763803 PMC10535830
